# Executive Functions Profile in Extreme Eating/Weight Conditions: From Anorexia Nervosa to Obesity

**DOI:** 10.1371/journal.pone.0043382

**Published:** 2012-08-21

**Authors:** Ana B. Fagundo, Rafael de la Torre, Susana Jiménez-Murcia, Zaida Agüera, Roser Granero, Salomé Tárrega, Cristina Botella, Rosa Baños, Jose M. Fernández-Real, Roser Rodríguez, Laura Forcano, Gema Frühbeck, Javier Gómez-Ambrosi, Francisco J. Tinahones, Jose C. Fernández-García, Felipe F. Casanueva, Fernando Fernández-Aranda

**Affiliations:** 1 Department of Psychiatry, University Hospital of Bellvitge-IDIBELL, Barcelona, Spain; 2 CIBER Fisiopatología Obesidad y Nutrición (CIBERObn), Instituto Salud Carlos III, Spain; 3 Human Pharmacology and Clinical Neurosciences Research Group, Neuroscience Research Program, IMIM-Hospital del Mar Research Institute, Parc de Salut Mar, Barcelona, Spain; 4 Department of Clinical Sciences, School of Medicine, University of Barcelona, Spain; 5 Laboratori d’Estadística Aplicada, Departament de Psicobiologia i Metodologia, Universitat Autònoma de Barcelona, Spain; 6 Department of Basic Psychology, Clinic and Psychobiology, University Jaume I, Castellón, Spain; 7 Department of Pyschological, Personality, Evaluation and Treatment, University of Valencia, Spain; 8 Service of Diabetes, Endocrinology and Nutrition, Institut d’Investigació Biomèdica de Girona (IdlBGi) Hospital Dr Josep Trueta, Girona, Spain; 9 Department of Endocrinology, University of Navarra, Pamplona, Spain; 10 Service of Diabetes, Endocrinology and Nutrition, Hospital Clínico Universitario Virgen de Victoria, Málaga, Spain; 11 Endocrine Division, Complejo Hospitalario U. de Santiago, Santiago de Compostela University, Spain; Federal University of Rio de Janeiro, Brazil

## Abstract

**Background:**

Extreme weight conditions (EWC) groups along a *continuum* may share some biological risk factors and intermediate neurocognitive phenotypes. A core cognitive trait in EWC appears to be executive dysfunction, with a focus on decision making, response inhibition and cognitive flexibility. Differences between individuals in these areas are likely to contribute to the differences in vulnerability to EWC. The aim of the study was to investigate whether there is a common pattern of executive dysfunction in EWC while comparing anorexia nervosa patients (AN), obese subjects (OB) and healthy eating/weight controls (HC).

**Methods:**

Thirty five AN patients, fifty two OB and one hundred thirty seven HC were compared using the Wisconsin Card Sorting Test (WCST); Stroop Color and Word Test (SCWT); and Iowa Gambling Task (IGT). All participants were female, aged between 18 and 60 years.

**Results:**

There was a significant difference in IGT score (F(1.79); p<.001), with AN and OB groups showing the poorest performance compared to HC. On the WCST, AN and OB made significantly more errors than controls (F(25.73); p<.001), and had significantly fewer correct responses (F(2.71); p<.001). Post hoc analysis revealed that the two clinical groups were not significantly different from each other. Finally, OB showed a significant reduced performance in the inhibition response measured with the Stroop test (F(5.11); p<.001) compared with both AN and HC.

**Conclusions:**

These findings suggest that EWC subjects (namely AN and OB) have similar dysfunctional executive profile that may play a role in the development and maintenance of such disorders.

## Introduction

Eating disorders (ED) and obesity appear to show parallel patterns in animal [1], [2] and human studies [3], [4]. Consistently, both ED and obesity have shown not only lifetime co-occurrence [5], but also common biological and environmental risk factors [6]–[8], as well as neurocognitive vulnerabilities [9], [10].

According to *continuum hypothesis*, ED and obese patients may share certain neurobiological correlates and neural/CNS circuitry pathways related to food reward system and just vary on how frequent or severe these traits are [11], [12]. The identification of common phenotypic features in ED and obesity like recurrent overeating episodes, commonly of highly palatable food, in relation to negative emotions and/or dietary restriction [13]–[15], have led to model these conditions as the consequence of an addiction to these food [16], [17]. However, up to now the strongest support for a model of food addiction arrives from animal models and further studies in humans seem to be crucial [18].

Neuroimaging studies in humans support the concept that alterations in dopamine circuits (mainly the mesocorticolimbic circuit) are implicated in both, eating behaviors and drug abuse [1], [19]. Based on these studies and animal model results, a model of food intake has been established [10], [20]. According to the authors, overeating is a sign of a disparity among circuits related to motivation and behavior (involving reward) and those implicated on inhibition response. Consistent with this theory, four brain circuits have been described: *reward–saliency*; *motivation–drive*; *learning–conditioning*; and *inhibitory control–emotional regulation–executive function* [20]. It is postulated that, in vulnerable subjects, the ingestion of elevated amounts of food can disturb the balance between such circuits, increasing the reinforcing value of food and reducing the activity of the circuits related to control and inhibition, which might result in an impulsive behavior and compulsive food ingestion. Interestingly, not only overeating but also under eating can disrupt dopamine brain reward systems [21]. Anorexia nervosa (AN) patients have also shown some traits of addiction [17], [21] as behaviors such as increases in restriction food intake and exercise can interfere with their day to day activities as, more or less, do substance abuse or dependence. In this regard, in a recent study [22] a mirror genetic effect was observed in extreme eating/weight conditions, demonstrating that carrying the 16p11.2 duplication confers a high risk for underweight, but also that reciprocal duplication at this locus is associated with being underweight. Thus, examining these disorders for their similarities and differences, not just in behaviors but also in their underlying biological and cognitive profiles, will hopefully tell us more about what is really going on and what really causes such unhealthy extreme weight conditions (EWC).

Neuropsychological studies in EWC support the hypothesis of an alteration on the *inhibitory control–emotional regulation–executive function* circuit. In general terms, and although EWC have been associated with difficulties in different cognitive variables [23]–[27], a core cognitive trait appears to be executive dysfunction, with a focus on three distinct neurocognitive constructs: decision making, response inhibition and cognitive flexibility [28]–[31]. AN, for instance, has been consistently associated with alterations on attentional and executive functioning (mainly set shifting and decision making) [32]–[41]. Interestingly, some aspects of executive functioning, such as cognitive flexibility, have been considered as a risk indicator and are believed to be a possible endophenotype in AN [35]. A poor executive function performance has also been described in obesity [42]–[44] with some relevant characteristics, such as impulsivity and reduced decision making abilities, resulting in inadequate self-control [42]–[44]. Particularly, it has been demonstrated that obese subjects show deficits in decision making assessed by Iowa Gambling Task (IGT) [42]–[44]. According to these authors, the performance of OB participants was as poor as the performance of drug users, observed in previous studies [44]. These results suggested a significant deficit on decision making associated with obesity and, once again, point to overeating palatable food as addiction-like behavior. Impulsivity, relating this inappropriate sensitivity to punishment, has also been observed in obese subjects corroborating an executive dysfunctional profile in obesity [43].

Such impairments on decision making, response inhibition and cognitive flexibility among EWC emphasizes the significance of an appropriate executive functioning for satisfactory control of eating behavior. Latest categorizations of executive functions have proposed that executive tasks vary in their motivational implication, with motivationally relevant tasks considered as “hot" and more abstract tasks considered as “cool" [45]. According to this hypothesis, EWC might be characterized by alterations in both hot (i.e. decision making) and cool (i.e. cognitive flexibility and inhibition control) executive functions. This is particularly important as it implicates different prefrontal brain circuits, including dorsolateral prefrontal cortex, anterior cingulated cortex and orbitofrontal cortex, on the dysfunctional executive profile observed in EWC subjects [46]. Consequently, differences between individuals in these areas would be making them more or less vulnerable to EWC. However, and in spite of the importance of straight comparison of the executive profiles in EWC subjects, research has explored each group separately and usually using single cognitive tasks [32], [34], [39]–[41], [43], [44]. As far as we know, only one study has compared cognitive performance in AN and obese subjects, that also included bulimia nervosa (BN) patients [42]. As expected, AN, BN and obese subjects performed significantly worse than healthy controls in a decision making task. However, the small sample size and the fact that only one cognitive function was evaluated somehow limit the result’s generalization.

To our knowledge, no comprehensive research covering the executive functioning profile of EWC has been done to date. Thus, the idea behind the study was to explore the integrity of the *inhibitory control–emotional regulation–executive function* circuit in EWC (from AN to obesity) while investigating if AN and obesity display a *continuum* dysfunctional executive profile (decision making, response inhibition and cognitive flexibility), in comparison with HC. It is postulated that both clinical groups should have similar executive profile, although the severity of the dysfunction should differ between them. As far as we know, this is the first study comparing EWC in their executive functions profile employing well-validated measures of decision-making, response inhibition and impulsivity.

## Methods

### Sample

All participants were informed about the research procedures and gave their informed consent in writing. Procedures were approved by the Ethical Committee of each of the aforementioned institutions. Seven centers from six Spanish sites (all involved in the CIBERobn Spanish Research Network) participated: the Eating Disorders Unit (Department of Psychiatry, University Hospital of Bellvitge-IDIBELL, Barcelona), the Department of Endocrinology at the University Hospital of Santiago (Santiago de Compostela); the Department of Diabetes, Endocrinology and Nutrition (Clinic University Hospital Virgen de Victoria, Málaga); the Department of Endocrinology (University of Navarra, Pamplona); the Diabetes, Endocrinology and Nutrition Department, Biomedical Research Institute of Girona (IdIBGi-Doctor Josep Trueta Hospital, Girona); the Hospital del Mar Research Institute (IMIM-Hospital del Mar, Barcelona) and the department of Basic Psychology, Clinic and Psychobiology (University Jaume I, Castellón). Enrolment into the study was between January 2010 and September 2011 and all consecutive patients referred to these institutions were included.

The total sample comprised 224 participants [35 AN patients, 52 obese subjects (OB) and 137 healthy controls (HC)]. All participants were female, aged between 18 and 60 years and spoke Spanish as their first language. AN patients were diagnosed by experienced clinicians (according to DSM-IV-TR diagnostic criteria) [47], by means of the structured clinical interview for DSM IV Axis I disorders (SCID-I) [48]. The interviewers were trained in the administration of these instruments.

The exclusion criteria in the clinical cases groups (AN and OB) include: (1) History of chronic medical illness or neurological condition that might affect cognitive function; (2) Head trauma with loss of consciousness for more than 2 min, learning disability or mental retardation; (3) Use of psycho-active medications or drugs (4) Being male; (5) Age under 18 or over 60 (to discard neuropsychological deficits associated with the age); (6) obese patients who have comorbid binge eating disorder (DSM-IV criteria).

Healthy controls were recruited through several sources including word-of-mouth and advertisements in the local university. Prior to assessment, HC were asked about lifetime or current presence of an ED or obesity. The lifetime history of health or mental illnesses profile was based on the general health questionnaire (GHQ)-28. Exclusion criteria in the HC group were: (1) Individuals who have suffered lifetime disorder of Axis I mental disorder; (2) Aged under 18 and over 60 years; and (3) Lifetime obesity (IMC>30).

### Procedures and Assessment

All participants underwent a comprehensive neuropsychological and clinical assessment. Weight and BMI on the day of assessment were measured for all subjects. The neuropsychological tests were selected to cover various aspects of executive functions including decision making, response inhibition, strategic planning and cognitive flexibility and were administered by a trained psychologist in a single session and in a randomized order.

### Neuropsychological Measures

All participants were assessed with the following neuropsychological tests: (a) The Wisconsin Card Sorting Test (WCST; [49]) (b) The Stroop Color and Word Test (SCWT; [50] (c) The Iowa Gambling Task (IGT; [51]).

#### (a) WCST

The WCST is a classical measure of planning capacity, cognitive flexibility, capacity of shifting among stimulus, and control of impulsive responses not aimed at achieving an objective. Subjects have to match a target card with one of four category cards: a single red triangle, two green stars, three yellow crosses, and 4 blue circles. Cards might be matched by color, number, or shape. After each trial a feedback is given to the participant, indicating if they have matched the card appropriately. However, along the task the classification rule is unpredictably changing. The test ends when the participant has completed 6 categories or 128 trials.

#### (b) SCWT

This paper and pencil test has shown adequate reliability, and construct validity for the assessment of inhibition and switching skills. The SCWT measures interference control, flexibility and attention. The task included three pages: (1) a page with color words printed in black ink; (2) a page with “Xs" printed in color; (3) a page with names of colors printed in an incongruent color (i.e. word “blue" printed in red ink). Participants have 45 seconds to reading as many words as possible in the first page and name the ink in pages 2 and 3. Three scores are obtained after completed the task: number of words (page 1), number of color-named “X" (page 2) and number of color-named words (page 3). An additional “interference score" is obtained. Higher scores in this variable indicate better capacity of inhibition response.

#### (c) IGT

This computer task evaluates decision-making, risk and reward and punishment value. The subject has to select 100 cards from four decks (A, B, C and D). After each card selection an output is given: gain or a gain and loss of money. Two decks (A and B) are not advantageous as the final loss is higher than the final gain. Decks C and D, however, are advantageous since the punishments are smaller. The final objective of the task is to make the most of profit and gain as much money as possible. This test is scored by subtracting the amount of cards selected from decks A and B from the amount of cards selected from decks C and D. Higher results point to better performance, while negative results point to preference for the not advantageous decks.

### Psychopathological Measures

#### (a) Eating Disorders Inventory 2 [52]

This is a reliable self-report questionnaire for evaluating some cognitive and behavioral traits that characterized eating disorders. The questionnaire includes 64 items from the EDI which are divided into eight scales: Drive for Thinness, Bulimia, Body Dissatisfaction, Ineffectiveness, Perfectionism, Interpersonal Distrust, Interoceptive Awareness and Maturity Fears. Twenty seven items were also added divided into three new scales: Asceticism, Impulse Regulation, and Social Insecurity. Each item is answered using a 6-point Likert scale and the answers are converted to standardized subscale scores.

#### (b) Symptom Checklist-90- Revised [53]

The SCL-90-R is a self-report questionnaire that measures a wide range of psychological and psychopathological symptoms and includes 90 items. Nine primary symptom dimensions could be obtained: Somatization, Obsession-Compulsion, Interpersonal Sensitivity, Depression, Anxiety, Hostility, Phobic Anxiety, Paranoid Ideation and Psychoticism. Additionally, three global indices are obtained: a Global Severity Index (GSI), used for evaluating the general psychological distress and considered as a summary of the test; a Positive Symptom Distress Index (PSDI), used for evaluating the intensity of symptoms; and a Positive Symptom Total (PST).

#### (c) Barratt Impulsiveness Scale (BIS) version 11 [54]

The BIS version 11 is a 30-item self-report questionnaire that measures impulsivity using three subscales including Attentional, Non planning, and Motor, as well as providing a total score. This scale quantifies personality factors relating to impulsivity using three subscales to measure impulsivity: “Attentional" (cognitive instability and inattention), “Non planning" (intolerance of cognitive complexity and lack of self-control), and “Motor" (lack of perseverance and motor impulsiveness). These subscales can be combined to form a total score. Each subject was asked to answer questions that measure some of the ways in which he or she acts or thinks. These responses are scored 1 through 4 with 4 reflecting the most “impulsive" response. Total scores can range from 30 to 120.

### Statistical Analysis

Statistical analyses were carried out with SPSS 19 for Windows (SPSS System; SPSS, Chicago, IL). First, analysis of variance (ANOVA) adjusted by the covariates age and education (measured as the number of years of completed studies) valued the association between the diagnosis subtype and the neurocognitive measures. Polynomial contrasts explored the presence of trends (linear and quadratic). Second, multiple regression models also adjusted by age and education valued the predictive accuracy of the BMI (considered as a continuous, kg/m2) on the neurocognitive measures. Global predictive accuracy was valued with R^2^ coefficients.

## Results

### Sample Characteristics

The HC group differed from the AN and OB groups with respect to years of education (p<.001) and age (p<.001) (See Table 1), which were controlled for in subsequent analyses as covariates. Group differences in BMI were as estimated (AN<HC<OB) (p<.001) (Table 1). As expected, the AN and OB groups differed from the HC on all of the EDI-2 subscales (p<.001) (except for perfectionisms in OB group) (See Table 2). Accordingly, general psychopathology, measured by means of SCL-90R scores, were higher for the AN and OB groups, when compared with the HC group (p<.001) (Table 2). Finally, OB subject demonstrated higher impulsivity compared with AN and HC on the BIS total score (p<.01) (Table 2).

**Table 1:** Sociodemographics.

					Contrasts (p-value)		
	Anorexianervosa(n = 35)	Healthy Controls (n = 137)	Obese(n = 52)	Group*p-value*	AN vs. HC	AN vs. OB	HC vs. OB
Age (yrs.); *mean (SD)*	28.1 (8.2)	24.8 (7.0)	40.5 (11.1)	**<.001**	.105	<.001	<.001
Civil status; *%*Single	75.0%	72.9%	18.9%	**<.001**	.929	<.001	<.001
Married	21.4%	24.1%	67.4%				
Separated-divorced	3.6%	3.0%	13.5%				
Studies level; *%* Primary	35.7%	6.9%	53.1%	**<.001**	<.001	.100	<.001
Secondary	39.3%	63.1%	40.6%				
High (university)	25.0%	30.0%	6.3%				
Education (yrs.); *mean (SD)*	14.5 (2.0)	15.5 (1.8)	14.3 (2.0)	**<.001**	.019	.943	.002
BMI (current); *mean (SD)*	17.2 (1.4)	21.5 (2.7)	39.8 (7.4)	**<.001**	<.001	<.001	<.001
BMI (maximum); *mean (SD)*	22.0 (2.8)	22.4 (2.8)	40.9 (8.3)	**<.001**	.931	<.001	<.001
BMI (minimum); *mean (SD)*	15.7 (2.2)	19.1 (2.0)	25.4 (3.9)	**<.001**	<.001	<.001	<.001

BMI: body mass index (kg/m^2^);

SD: standard deviation.

**Table 2:** Psychometrical characteristics among the groups.

	Mean (standard deviation)			ANOVA adjusted by age and studies					
	Anorexia nervosa(n = 35)	Healthy Controls(n = 137)	Obese (n = 52)	Diagnose	Contrasts: polynomial (p) andpost-hoc comparison (MD)				
				*p*	Lineal	Quadratic	AN vs. HC	AN vs. OB	OB vs. HC
EDI: Drive for thinness	12.2 (7.59)	2.56 (4.15)	11.2 (5.46)	**<.001**	.297	<.001	9.453*	1.370	8.083*
EDI: Body dissatisfaction	12.8 (9.02)	5.31 (6.10)	18.3 (6.95)	**<.001**	<.001	<.001	7.815*	−6.705*	14.519*
EDI: Interoceptive awareness	8.00 (6.25)	1.42 (2.51)	5.62 (5.86)	**<.001**	.014	<.001	6.273*	2.582*	3.691*
EDI: Bulimia	3.09 (3.60)	.63 (1.62)	2.49 (2.98)	**<.001**	.360	<.001	2.430*	.550	1.880*
EDI: Interpersonal distrust	5.15 (5.16)	1.81 (2.72)	4.10 (4.09)	**<.001**	.237	<.001	3.316*	1.063	2.252*
EDI: Ineffectiveness	7.61 (6.25)	1.43 (2.04)	5.54 (4.76)	**<.001**	.011	<.001	5.836*	2.389*	3.447*
EDI: Maturity fears	6.94 (5.76)	3.57 (3.27)	6.90 (4.49)	**<.001**	.738	<.001	3.255*	.335	2.920*
EDI: Perfectionism	5.42 (5.03)	3.75 (3.11)	3.31 (3.13)	**.013**	.035	.071	2.023*	1.884*	.139
EDI: Impulse regulation	4.24 (5.12)	1.11 (2.34)	2.74 (4.53)	**<.001**	.247	<.001	3.332*	1.011	2.321*
EDI: Ascetism	5.45 (4.76)	1.84 (1.95)	5.49 (4.10)	**<.001**	.979	<.001	3.750*	−.020	3.770*
EDI: Social insecurity	5.36 (4.71)	1.75 (2.38)	4.46 (4.15)	**<.001**	.131	<.001	3.358*	1.250	2.108*
EDI: Total score	76.2 (43.7)	25.2 (18.8)	70.1 (30.7)	**<.001**	.406	<.001	50.840*	5.709	45.131*
SCL90-R: GSI score	1.43 (.78)	.57 (.42)	1.18 (.72)	**<.001**	.021	<.001	.808*	.330*	.478*
SCL90-R: PST score	55.9 (19.0)	32.5 (18.6)	50.4 (23.8)	**<.001**	.111	<.001	21.768*	8.070	13.698*
SCL90-R: PSDI score	2.12 (.65)	1.44 (.32)	1.92 (.54)	**<.001**	.024	<.001	.656*	.253*	.403*
BIS: cognitive	12.79 (5.08)	12.65 (4.52)	14.38 (3.84)	**.030**	.035	.040	.614	−2.507*	3.122*
BIS: motor	13.65 (6.08)	13.67 (5.62)	16.57 (7.09)	.197	.088	.345	−.217	−2.789	2.573
BIS: no plan	13.21 (5.29)	16.12 (6.36)	18.30 (6.29)	**.005**	.003	.506	−3.267*	−4.891*	1.624
BIS: total score	39.65 (11.5)	42.44 (12.8)	49.24 (12.5)	**.010**	.002	.379	−2.869	−10.187*	7.318*

MD: mean difference (contrast value);

* Significant contrast (.05 level);

EDI: Eating Disorders Inventory;

SCL90R: Symptom Checklist-90;

BIS: Barratt Impulsiveness Scale.

### Neuropsychological Assessment

**Figure 1:**
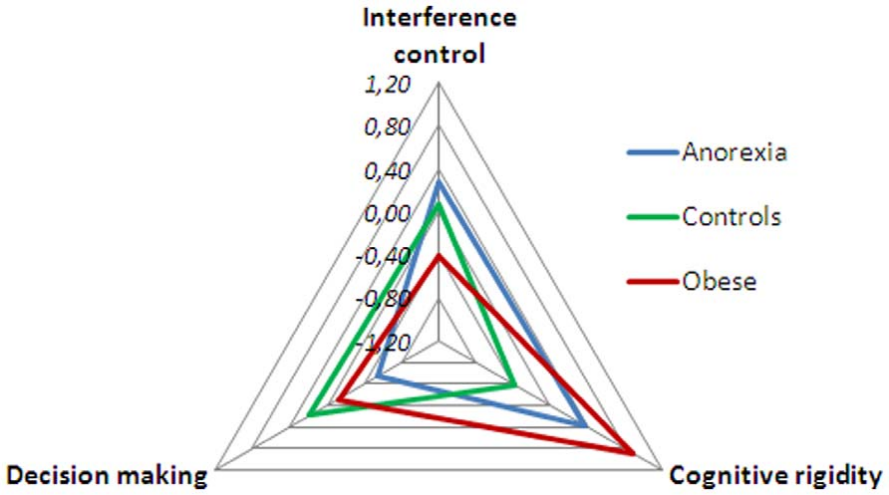
Radar chart illustrating the performance of the Anorexia nervosa, Obese and Healthy eating/weight control groups for interference control, cognitive rigidity and decision making.

Data on neuropsychological test performance of the three groups are presented in Table 3 and Figure 1. Results showed that OB displayed a worse performance in Stroop interference score, comparing with both AN patients and HC. The IGT total (p<.001) and almost all sub-scores (p<.01) were significantly lower for AN patients and OB subjects relative to the HC participants (Table 3). The AN and OB mean performance on the WCST was significantly worse than that of HC. Both global score (p<.001) and number of errors (p<.001) (perseverative and non-perseverative) were significantly higher in the clinical groups (Table 3). Accordingly, as shown in Figure 1, a radar chart represents graphically this linearity and how the three groups performed across the three neuropsychological domains: cognitive flexibility/rigidity (WCST), response inhibition (SCWT), and decision making (IGT). The data were converted to z scores, to allow us having similar measures for all the variables used.

**Table 3:** Comparison of neurocognitive measures between diagnosis subtypes.

	Mean (standard deviation)			ANOVA adjusted by age and studies					
	Anorexia nervosa(n = 35)	Healthy Controls (n = 137)	Obese (n = 52)	Diagnose	Contrasts: polynomial (p) andpost-hoc comparison (MD)				
				*p*	Lineal	Quadratic	AN vs.HC	AN vs.OB	OB vs.HC
**STROOP**									
Interference	7.9 (9.4)	6.2 (7.5)	2.3 (7.6)	.133	.049	.846	2.267	3.995*	−1.729
**IGT**									
Block 1	−2.2 (4.5)	−1.8 (6.8)	−0.4 (7.2)	.966	.862	.876	−.321	−.290	−.030
Block 2	−1.3 (4.9)	2.5 (8.2)	0.4 (8.0)	**.007**	.607	.002	−3.574*	.976	−4.549*
Block 3	−0.4 (5.5)	5.3 (8.1)	3.1 (8.6)	**.001**	.823	<.001	−5.195*	−.438	−4.757*
Block 4	−0.2 (8.5)	5.6 (9.2)	2.5 (8.6)	**.009**	.310	.008	−5.292*	−2.333	−2.959
Block 5	−1.0 (9.7)	5.0 (10.1)	2.1 (9.2)	**.002**	.288	.021	−5.335*	−2.707	−2.628
Total	−5.1 (23.0)	16.5 (28.8)	7.7 (30.1)	**.001**	.493	<.001	−19.717*	−4.792	−14.924*
**WCST**									
Total trials	99.1 (23.6)	83.6 (14.5)	109.8 (21.3)	**<.001**	.695	<.001	12.479*	−1.753	14.233*
Correct response	67.4 (12.5)	68.0 (7.0)	72.5 (13.8)	.305	.127	.631	−1.129	−3.907	2.778
Total errors	31.7 (25.9)	15.6 (13.5)	37.2 (23.0)	**<.001**	.643	<.001	13.608*	2.153	11.455*
Perseverative responses	17.0 (14.9)	8.3 (6.6)	22.6 (18.7)	**.001**	.797	<.001	7.209*	−.756	7.965*
Perseverative errors	15.0 (12.1)	7.9 (5.9)	19.9 (14.7)	**.001**	.731	<.001	5.773*	−.826	6.599*
NO perseverative errors	16.7 (17.2)	7.7 (8.2)	17.4 (12.4)	**.001**	.296	.001	7.835*	2.979	4.855
CLR	58.0 (17.8)	64.5 (9.4)	62.1 (18.7)	.072	.113	.175	−6.014*	−5.597	−.417

IGT: Iowa Gambling Task;

WCST: Wisconsin Card Sorting Test;

CLR: Conceptual Level Response;

MD: mean difference (contrast value);

* Significant contrast (.05 level).

Finally, no significant differences on neuropsychological performance as a function of BMI were observed [Stroop interference (p = 0.08); IGT Total (p = 0.64); WCST Total trials (p = 0.12); WCST Total errors (p = 0.64); WCST Perseverative errors (p = 0.34); WCST no perseverative errors (p = 0.98); WCST Perseverative responses (p = 0.34); WCST CLR (p = 0.11)].

## Discussion

This study set out to examine whether EWC (from Anorexia nervosa to Obesity) display a similar profile of executive dysfunction. Results showed a similar pattern of abnormal scores in EWC populations across the executive domains assessed by the neuropsychological tasks. Group differences were more marked on the cognitive flexibility and decision making domains, although the inhibition response task also showed marginally statistical differences. Therefore, cognitive results are in agreement with several previous neuropsychological studies showing that EWC deficits extend to several executive processes [32], [33], [36], [37], [39]–[41], [44], [55], [56], almost certainly relying on the functioning of different fronto striatal systems, including the dorsolateral prefrontal cortex and the orbitofrontal cortex [21], [57]–[59].

Our results support the hypothesis that decision making ability is impaired in EWC. Different profiles in IGT performance were found between groups: HCs performed better and learned to keep away from not advantageous decks, while the performance of AN and OB subjects did not improve along the task. Both, AN and OB subjects go for choices that result in elevated immediate gains despite important future losses, showing a similar level of impairment between them. Therefore, according to previous general conducted studies [32], [39], [40], [42]–[44], [55], [56], the extreme eating/weight behaviors of these subjects might partially be an expression of their incapacity to successfully regulate reward and punishment, which might be translated into deficit in planning every day functioning. From a clinical perspective, it might be postulated that there are reasonable similarities between their test performance and their day to day pathological eating behaviors.

However, the cognitive mechanism underlying the decision making performance in AN and OB subjects might be different. Obesity performance might be associated with an elevated level of impulsivity, which has also been observed in their self-administered questionnaire (BIS) and Stroop test results. It has been established that impulsive subjects have marked limitations for learning suitable associations between reward and punishment [60], [61]. As a consequence, there is a tendency for these subjects to have a reduced capacity to delay gratification, showing a reward-based impulsivity which characterizes overeating behaviors and weight gain. On the other hand, the cognitive mechanism explaining decision-making profile in AN patients might be somewhat divergent, characterized by a rigid behavior. In contrast to obese subjects, clinical reports document perseverative, obsessive, and rigid thinking styles in patients with AN [62], [63]. As a general rule, these patients show an elevated resistance to change [63]. Thus, persistence with similar answers when the way was to provide an alternative and more efficient response might be explaining the cognitive decision making picture of AN patients, where features such as rigidity, perfectionism and compulsive behaviors are being present.

Concerning the WCST’s performance our results are consistent with previous research done in both AN and obese subjects [33]–[35], [37], [41], and also point to a similar cognitive profile between them. EWC subjects performed significantly less well than HC on this task, showing less abstraction ability and flexibility of thought compared with the HC group. They are capable of acquiring the first rule but are unable to adjust their behavior after the rule change. In other words, they have difficulties while switching between different rules or when the development of new rules is needed, observed by their significantly elevated number of errors. Thus, an important conclusion of our study is that WCST’s performance appears as a further potential EWC-associated intermediate phenotype. However, it is important to considerer that the WCST is a multifactorial and complex test that draws on additional cognitive functions beyond set-shifting and maintenance [64]. Performance in this task might be compromised due to different reasons, such as deficits in memory, sustained attention and response suppression to irrelevant material. Consequently, it would be interesting to more clearly elucidate specific error patterns in WCST performance and thus clarify the underlying cognitive operations. To this end, WCST analogues, such as Extra-Dimensional Intra-Dimensional set shift tasks (CANTAB), might be applied.

Finally, only the obese group showed deficiencies in attention and performance during the Stroop test, suggesting that adult obese subjects respond more impulsively and make more errors on an interference control task. The Stroop task implicated the capacity to choose a weaker but task-relevant answer, regardless a stronger, but task-irrelevant one [65]. This process is thought to be critical for suppression of inappropriate/unwanted actions that can interfere with achieving motor, cognitive, or emotional goals [66]. Our results not only confirmed the hypothesis that obese persons have difficulty inhibiting automatic or dominant behavior and intrusive thoughts [67], [68], but also point to inhibition response as a distinguishing trait of obesity among EWC executive profile.

The executive deficits observed are not likely to be related to starvation, as shown by the lack of correlation between neurocognitive functioning and BMI score. On the contrary, our results are consistent with the *continuum hypothesis*: subjects with either excessive food intake or food restriction show an analogous dysfunctional executive profile [12]. We hypothesized that EWC populations might have a similar dysfunctional neural pattern in brain circuits related with reward and executive functioning, mainly decision making and flexibility. Functional brain imaging studies in healthy volunteers suggest that this pattern of deficits undoubtedly involves several brain regions, including mesocorticolimbic systems, although the prefrontal cortex seems to be particularly relevant [69], [70].

Before taking behavioral actions, the prefrontal cortex control the processing in different parts of the brain, including subcortical and limbic reward areas, activating circuits that manage with current task demands, but according to our goals [71], [72]. That is why the prefrontal cortex is decisive in situations when the neural patterns associated with some inputs and behaviors are softly established compared with others (Stroop task), are quickly changing (WCST), or the external situation lead us to make appropriate decisions giving up immediate benefit (IGT). Without a correct PFC activity, the most frequently used cerebral pathways could always prevail or, if these do not exist, behavior could be random. Under such circumstance the behavior might be impulsive, inappropriate, and rigid, as observed in EWC and confirm by means of our results. Thus, deficits on flexibility, decision-making and inhibition response observed in EWC might be no more than a behavioral expression of a dysfunctional prefrontal circuit’s activity.

Our findings are also supported by limited neuroimaging data in EWC adults, which point to dysfunction on the fronto-subcortical circuits in such patients [57], [73], [74]. For instance, decreases in perfusion and metabolism have been observed in frontal regions in AN patient and obese subjects, mainly in the superior frontal cortex, dorsolateral PFC (DLPFC) and orbitofrontal cortex (OFC) [57], [73], [74]. It has also been demonstrated decreased activation in anterior cingulate cortex and striatum associated with impaired cognitive-behavioral flexibility in patients with anorexia nervosa [57], [74]. However, few brain imaging studies have been conducted to date, and further studies are needed in order to confirm the impairment on brain fronto-subcortical circuits in EWC.

Our study has several important strengths including the relatively large sample size. Furthermore, most of the previous studies on the topic had been conducted on AN patients or obese subjects separately and generally using only one cognitive task. Conversely, our study was specifically designed to test the common executive dysfunction on these populations, by using three well validated executive tests. However, the results of this study should be interpreted in the context of some limitations. First, measures of intelligence quotient (IQ) were not considered, which might have influenced group differences, considering that greater scores of this variable might be related with better executive profile. However, years of education, as a cognitive level measure have been considered on the statistical analysis. Second, the obese subjects were significantly older than the AN patients and healthy controls, although differences in age were statistically controlled. In addition, only females were included in the study, thus the results are not applicable to males and replication with a group including males should be considered. Future studies should consider including additional decision-making, inhibition response and cognitive flexibility tasks in order to better understand such cognitive variables and to shed light on how they work. Measures of working memory and planning can also be included, as some studies including a prospective longitudinal approach have demonstrated alteration in those executive domains in AN patients [75]. The relation to mentalizing tasks and autism spectrum disorder in AN patients can also be considered for evaluation [75].

In addition, the role of some biomarkers, such as hormones levels, on cognitive functions should also be taken into consideration. Some studies suggest that adipokines, and principally leptin, may influence the pathogenesis of dementia [76]. Higher leptin levels have been related to a lower incidence of dementia, which is clearly associated with a dramatic cognitive decline [76]. Higher leptin levels are also associated with increased gray matter volume in the hippocampus and cerebellum and with higher total brain volume assessed with MRI [76], [77]. The main determinant of leptin levels is adipose tissue mass, and hyperleptinemia has been linked with obesity [78]. Thus, considering the role of leptin on cognition and brain functioning, and its association with BMI, the study of its implication on EWC seems to be relevant. Finally, more studies using cognitive and brain imaging approaches are needed to better comprehend how cerebral circuits related to executive functions are working in EEC, and also to consider some confounding variables such as vascular brain lesions, specially in obese subjects. In this regard, according to the vascular hypothesis of dementia, obesity might be considered a midlife vascular risk factor associated with both cerebrovascular lesions and a higher cognitive decline [79]. Considering all of these variables might be useful while deciding on a suitable clinical intervention.

To our knowledge, this is the first study that investigates executive functioning in EWC populations. The finding that both, AN patients and obese subjects, exhibit similar difficulties in decision making and cognitive flexibility has several implications. Even as different in their phenotype, EWC does share some cognitive alterations associated with abnormalities of different PFC functional systems. Particularly, they are believed to have commonalities of dysfunction in prefrontal circuitry that mediates executive functions, reward and behavioral regulation. In view of our results, a therapeutic individualized approach, focalized on psychological and cognitive interventions, in line with the cognitive and motivational profiles of the patient might offer significant help in improving healthy eating behaviors, as suggested by other authors [80], [81]. Therefore, in order to maximize clinical interventions effectiveness, the cognitive evaluation of AN patients and obese people have to consider the different aspects of the executive functions. In line with this assessment, treatment should focus on self-control problems, impulsive behaviors or decision making deficits that typify these disorders.
